# IQD1 Involvement in Hormonal Signaling and General Defense Responses Against *Botrytis cinerea*

**DOI:** 10.3389/fpls.2022.845140

**Published:** 2022-04-26

**Authors:** Omer Barda, Maggie Levy

**Affiliations:** Department of Plant Pathology and Microbiology, The Robert H. Smith Faculty of Agriculture, Food and Environment, The Hebrew University of Jerusalem, Rehovot, Israel

**Keywords:** *Botrytis cinerea*, defense responses, glucosinolates, hormone signaling, IQD1

## Abstract

IQ Domain 1 (IQD1) is a novel *Arabidopsis thaliana* calmodulin-binding protein, which was found to be a positive regulator of glucosinolate (GS) accumulation and plant defense responses against insects. We demonstrate here that the IQD1 overexpressing line (*IQD1*^OXP^**) was also more resistant also to the necrotrophic fungus *Botrytis cinerea*, whereas an IQD1 knockout line (*iqd1-1*) was much more sensitive. Furthermore, we showed that IQD1 is up-regulated by jasmonic acid (JA) and downregulated by salicylic acid (SA). A comparison of whole transcriptome expression between *iqd1-1* and wild type plants revealed a substantial downregulation of genes involved in plant defense and hormone regulation. Further examination revealed a marked reduction of SA and increases in the levels of ethylene, JA and abscisic acid response genes in the *iqd1-1* line. Moreover, quantification of SA, JA, and abscisic acids in *IQD1*^OXP^** and *iqd1-1* lines relative to the wild type, showed a significant reduction in endogenous JA levels in the knockout line, simultaneously with increased SA levels. Relations between *IQD1*^OXP^** and mutants defective in plant-hormone response indicated that IQD1 cannot rescue the absence of NPR1 or impaired SA accumulation in the NahG line. IQD1 cannot rescue *ein2* or *eto1* mutations connected to the ethylene pathway involved in both defense responses against *B. cinerea* and in regulating GS accumulation. Furthermore, IQD1cannot rescue the *aos*, *coi1* or *jar1*mutations, all involved in the defense response against *B. cinerea* and it depends on JAR1 to control indole glucosinolate accumulation. We also found that in the *B. cinerea*, which infected the *iqd1-1* mutant, the most abundant upregulated group of proteins is involved in the degradation of complex carbohydrates, as correlated with the sensitivity of this mutant. In summary, our results suggest that IQD1 is an important *A. thaliana* defensive protein against *B. cinerea* that is integrated into several important pathways, such as those involved in plant defense and hormone responses.

## Significance Statement

IQD1 is involved in glucosinolate accumulation and in general defense responses. JA activates IQD1 requires functional JA/ET and SA signaling pathways to control glucosinolate accumulation and defend against *Botrytis cinerea* and depends on JAR1 to control indole glucosinolate accumulation.

## Introduction

Plants must continuously adapt and protect themselves both against abiotic stressors, such as drought, extreme temperatures, improper lighting and excessive salinity and biotic stress imposed by other organisms such as viruses, bacteria, fungi and insects. Plants are resistant to most pathogens and in spite their sessile nature they have evolved a wide variety of constitutive and inducible defense mechanisms. Constitutive defenses include pre-formed physical barriers composing of cell walls, a waxy epidermal cuticle, bark and resins (Heath, 2000a). If this first line of defense is breached, then the plant must resort to a different set of chemical mechanisms in the form of toxic secondary metabolites and antimicrobial peptides, which are ready to be released upon cell damage (Tam et al., 2015). These pre-formed compounds are either stored in their biologically active forms like saponins (Podolak et al., 2010), or as precursors that are converted into toxic anti-microbial molecules only after pathogen attack, as exemplified by the glucosinolate-myrosinase system (Wittstock and Halkier, 2002). Other defense responses require detection of the invading pathogen by the plant and activation of inducible responses, often culminating in deliberate localized cell suicide in the form of the hypersensitive response (HR) so as to limit pathogen spread (Gilchrist, 1998; Heath, 2000b). Plants activate local defenses against invading pathogens within minutes or hours, with levels of resistance in distal tissue being influenced by systemic signals mediated by plant hormones. The identity of the pathogen determines the type of systemic response. The classic dogma holds that jasmonic acid (JA) and ethylene signaling activate resistance against necrotrophs whereas the salicylic acid (SA) signaling pathway is important for fighting biotrophic pathogens, although it also plays some role in defending against the necrotrophic fungi *Botrytis cinerea* (Govrin and Levine, 2000; Ferrari et al., 2003; Vuorinen et al., 2021). These two pathways are mostly antagonistic, with the balance of crosstalk between them affecting the outcome of the pathology (Glazebrook, 2005). *Botrytis cinerea* causes disease in more than 200 plant species including numerous economically important crops such as tomatoes and grapes (AbuQamar et al., 2016). The fungus has a predominantly necrotrophic lifestyle that involves killing plant host cells by diverse phytotoxic compounds and degrading enzymes, after which it extracts nutrients from the dead cells. It comprises nearly 300 genes encoding proteins considered Carbohydrate-Active enZymes (CAZymes) and selectively attacks cell wall polysaccharides, depending on the carbohydrate composition of the invaded plant tissue (Blanco-Ulate et al., 2014). Plant defense response against this pathogen are complex and involve many genes related to phytohormone signaling, including the ethylene, abscisic acid, JA, and SA pathways (Kliebenstein et al., 2005).

Glucosinolates (GSs) are sulfur-rich anionic secondary metabolites characteristic of the crucifers (the Brassicaceae family), with important biological and economic roles in plant defense and human nutrition. Currently, there are approximately 140 naturally produced GSs described in the literature (Nguyen et al., 2020). They all share a common chemical structure, consisting of a β-D-glucopyranose residue linked via a sulfur atom to a (Z)-N-hydroximinosulfate ester, as well as a variable R group. GSs are divided into three classes according to their precursor amino acid. Compounds derived from methionine, alanine, leucine, isoleucine or valine are called aliphatic GSs, those derived from phenylalanine or tyrosine are called aromatic GSs and those derived from tryptophan are called indole GSs. The various ecotypes of the model plant *Arabidopsis thaliana* produce about 40 different GSs of the indole and aliphatic families. GSs become biologically active only in response to tissue damage, at which point they are enzymatically cleaved by special thioglucoside glucohydrolases known as myrosinases. These enzymes hydrolyze the glucose moiety of the GS, creating an unstable aglycone that can rearrange to form nitriles, thiocyanates, isothiocyanates or other active products. To prevent damage to the plant itself, spatial compartmentalization separates myrosinases, which are mainly stored in specialized myrosin cells, from their GS substrates that are found in vacuoles throughout the plant cells (Halkier and Gershenzon, 2006). In recent years, it was demonstrated that GS metabolism is an important component of the plant defense response against fungi and other microbial pathogens (Bednarek et al., 2009; Clay et al., 2009; Buxdorf et al., 2013). The regulation of GS metabolism is a complex process involving all major plant defense hormones (i.e., SA, JA, abscisic acid, and ethylene) but also other hormones, such as gibberellic acid, brassinosteroids, and auxin are also involved (Mitreiter and Gigolashvili, 2021). Six R2R3-MYB transcription factors are known to be positive regulators of GS biosynthesis. Specifically, MYB28, MYB29, and MYB76 affect aliphatic GS (Li et al., 2013), whereas MYB34, MYB51 and MYB122 regulate indole GSs (Frerigmann and Gigolashvili, 2014a; Mitreiter and Gigolashvili, 2021).

IQD1 has also been found to be a positive regulator of GS accumulation and plant defense responses against insects (Levy et al., 2005). IQD1 is part of a family that comprises 33 IQD genes in *A. thaliana*, all encoding proteins possessing a distinct plant-specific domain of 67 conserved amino acids termed the IQ67 domain. The IQ67 domain is characterized by a unique and repetitive arrangement of IQ, 1-5-10 and 1-8-14 calmodulin recruitment motifs (Abel et al., 2005). IQD genes are not unique to *A. thaliana*, as bioinformatics and molecular tools have identified IQD genes in additional plant species, such as rice, tomato, soybean, grapevine, and others (Filiz et al., 2013; Huang et al., 2013; Feng et al., 2014; Ma et al., 2014; Cai et al., 2016; Wu et al., 2016; Yuan et al., 2019; Liu et al., 2020; Mei et al., 2021; Rehman et al., 2021).

IQD genes also play diverse roles in plants unrelated to glucosinolate synthesis or defense mechanisms. A set of microarray studies designed to identify DELLA responsive genes revealed *A. thaliana* IQD22 as one of several proteins involved in the early response to gibberellin (Zentella et al., 2007). SUN, the tomato IQD12 homolog was found to be a major factor controlling the elongated fruit shape of tomato fruits (Xiao et al., 2008). IQD family proteins from the cotton *Gossypium hirsutum* (GhIQD31 and GhIQD32) were found to induce drought and salt stress tolerance (Yang et al., 2019). A continuous body of work from the recent years has pointed to a general role for IQD proteins as microtubule-binding proteins that recruit calmodulin to subcellular compartments so as to coordinate plant development and cell shape formation (Bürstenbinder et al., 2013, 2017; Guo et al., 2020; Bao et al., 2021). Previous studies also identified the kinesin light chain-related protein-1 (KLCR1) as an IQD1 interactor in *A. thaliana* and demonstrated the association of IQD1 with microtubules. It was further suggested that IQD1 and related proteins provide scaffolds that facilitate cellular transport of RNA along microtubular tracks, as a mechanism to control and fine-tune gene expression and protein sorting, thus explaining the pleiotropic effects of IQD1 in many cellular pathways (Abel et al., 2013; Bürstenbinder et al., 2013). The *A. thaliana* IQD16 was also implicated as a microtubule-associated protein affecting cortical microtubule ordering, apical hook formation and cell expansion (Li et al., 2020). Indeed, a recent publication showed that the DUF4005 domain, frequently found in the C-terminal portion of IQD proteins, is a microtubule-binding motif (Li et al., 2021). In the current work, we sought to elucidate the mechanism of action of the *A. thaliana* IQD1 protein and define its involvement in hormone signaling and in basal defense against *B. cinerea*.

## Results

### IQD1 Expression Levels Correlate With *Botrytis cinerea* Resistance

**FIGURE 1:**
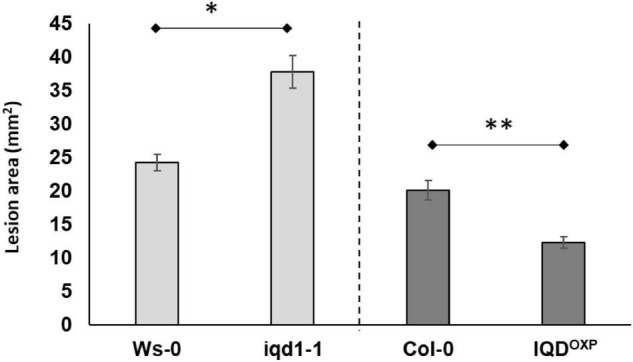
Pathogenicity of *B. cinerea* to Arabidopsis plants. Shown are averages of lesion size (mm^2^) of *B. cinerea* (Grape) on parental WT plants (*Ws-0* or *Col-0*), on an IQD1 knockout line iqd1*-1* and the over expressor line *IQD1*^OXP^** 72 h post-inoculation. Each column represents an average of 20 leaves, with standard error bars indicated. Asterisks above the columns indicate statistically significant differences at *P* < 0.05 from the corresponding WT, as determined using Student’s *t-*test. Results shown are from a biological replicate representative of six independent experiments.

Inoculation analysis with *B. cinerea* demonstrated that the *IQD1* enhancer trap line, which contains four repeats of the enhancer region of the constitutively active 35S promoter of *cauliflower mosaic virus* adjacent to *IQD1* gene (*IQD1*^OXP^**), was more resistant to the necrotrophic fungus, whereas an IQD1 knockout line (*iqd1-1*) was significantly more sensitive, both relative to wild type (WT) *A. thaliana* plants (Figure 1).

### Transcriptional Characterization of the IQD1 Knockout Line

#### Global Gene Expression Analysis of *iqd1-1* vs. Wild Type Plants

To evaluate the molecular changes underlying the impact of *IQD1* expression on defense responses, we performed global gene expression analysis using RNA samples from WT and *iqd1-1* rosette leaves 48 h after *B. cinerea* or mock inoculation.

A summary of parsed reads from each of the four samples of reads mapped to the *A. thaliana* genome is provided in Supplementary Table 1. Our analysis revealed that 48 h post-mock inoculation, a total of 3,508 genes were differentially expressed at least fourfold in *iqd1-1* knockout plants, as compared with WT *A. thaliana* (Supplementary Figure 1A and Supplementary Data Sheet 1). Among these genes, 1054 were up-regulated in mock-treated *iqd1-1* (and down-regulated in WT plants), yet more than double this number, i.e., 2,454 genes, exhibited down-regulation in the *iqd1-1* mutant (and expressed higher in the WT). Eighteen genes were selected for qRT-PCR analysis to validate the RNA-Seq data. These comprise 7 genes that were up-regulated in mock-treated *iqd1-1* vs. WT lines and 11 genes that were down-regulated in the same experiment. When expression ratios obtained by qRT-PCR were plotted vs. the respective RNA-Seq values, it was shown that the qRT-PCR results were in agreement with RNA-Seq data (Supplementary Figure 1B).

#### Functional Annotation of Differentially Expressed Genes

**FIGURE 2:**
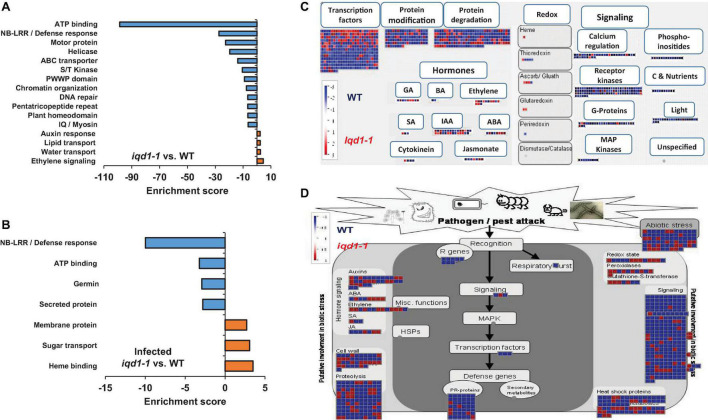
Differentially expressed clusters and genes in *iqd1-1* vs. WT plants. Enriched annotation terms of functional-related genes were grouped into clusters using the DAVID bioinformatics resources website. Positive enrichment scores denote upregulated clusters in *iqd1-1* lines, while negative values denote up-regulated clusters in WT plants. **(A)** Differentially expressed clusters and genes in *iqd1-1* vs. WT plants. **(B)** Differentially expressed clusters in infected *iqd1-1* vs. infected WT plants. **(C,D)**. A MapMan regulation overview map showing differences in transcript levels between *iqd1-1* and WT plants. Red squares represent higher gene expression in mock treated *iqd1-1* plants while blue squares represent higher gene expression in mock-treated WT plants, A regulatory network is presented in **(C)**, while stress response network is shown in **(D)**.

Functional annotation of our data revealed that there were many more significantly down-regulated than up-regulated clusters in the *iqd1-1* mutant. The down-regulated genes encode protein families that serve a wide array of functions, acting as molecular motors, DNA organization and repair proteins, trans-membrane transporters, and contributing to gene regulation and defense responses (Figure 2A). It is of note that the second most down-regulated cluster constitutes the nucleotide-binding domain leucine-rich repeat (NB-LRR) plant resistance genes. The products of these genes are involved in the detection and initiation of specific plant defenses against diverse pathogen groups. The fact that many NB-LRR genes are less expressed in the *iqd1-1* knockout plants may contribute to sensitivities of this lines to pests (Levy et al., 2005). The upregulated clusters in *iqd1-1* mainly comprise water and lipid transporters and ethylene signaling genes, although these presented lower enrichment scores than did the down-regulated clusters.

As demonstrated in Figure 2C, most of the genes assigned to plant cell regulation are down-regulated in *iqd1-1*, as compared to non-infected WT (Figure 2C, blue squares). These genes mainly encode transcription factors, proteins involved in protein modification and degradation, receptor kinases and hormone biosynthesis and signaling proteins. The only exception were ethylene-, JA- and abscisic acid (ABA)-signaling genes, which are mostly upregulated in *iqd1-1*, relative to WT plants (Figure 2C, red squares).

When we considered differentially expressed genes (DEGs) connected to biotic stress in *iqd1-1* vs. WT lines, we found that most of the genes responsible for plant defenses were down-regulated in the *iqd1-1* mutant (Figure 2D, blue squares), including genes encoding heat shock proteins, pathogenesis-related proteins, peroxidases and other stress response proteins. In light of the above, we can speculate that *iqd1-1* plants are impaired in sensing, signal transducing and responding to pathogen attacks. Furthermore, most of the 69 DEGs responsible for abiotic stress response are also downregulated in the *iqd1-1* mutant. These include genes for heat shock proteins, dehydration-responsive proteins and molecular chaperones, implying that the mutant presents an impaired response to abiotic, as well as biotic stressors. The list of affected genes, along with the fold change in their expression and descriptions of the functions of their products is provided in Supplementary Data Sheet 2.

#### Comparing *Botrytis cinerea*-Infected *iqd1-1* and Wild Type Plants

We found that 48 h post-inoculation with the necrotrophic fungi *B. cinerea*, 2,210 genes were upregulated and 3,129 genes were downregulated in infected WT plants, as compared to their mock-treated counterparts (Supplementary Figure 1A and Supplementary Data Sheet 3). Furthermore, 2,343 genes were upregulated and 3,092 were downregulated in infected *iqd1-1* plants, as compared to the mock-treated mutant plants (Supplementary Figure 1A and Supplementary Data Sheet 4). Using the DAVID web resource, it was revealed that extensive changes in gene expression occurred both in WT (Supplementary Figure 2) and in *iqd1-1* knockout plants (Supplementary Figure 3) after infection. In both cases, clusters comprising gene families that participate in photosynthesis were markedly down-regulated (i.e., negative values) upon infection, as the plant is tuned in to fight the invading pathogen. Up-regulated clusters (i.e., positive values) consist of genes for plant defense protein families.

Direct comparison of DEGs in infected *iqd1-1* vs. infected WT plants showed that 702 genes were up-regulated in the infected mutant, while 850 genes were up-regulated in infected WT plants (Supplementary Data Sheet 5). Analysis of our RNA-Seq results revealed that WT plants express more NB-LRR resistance genes and those encoding defensive cell-wall associated germin glycoproteins, which are induced upon pathogen recognition (Figure 2B, negative values). On the other hand, infected *iqd1-1* plants over-expressed heme-binding proteins and sugar transporters (Figure 2B, positive values).

### Involvement of IQD1 in Hormone Signaling and Glucosinolate Biosynthesis

#### Expression of Plant Hormone Related Genes in *iqd1-1* Plants

RNA-Seq transcriptional analysis of *iqd1-1* plants, as compared to WT, revealed substantial changes in gene expression in the mutant. Many of the DEGs are involved in hormone biosynthesis and responses (Table 1). Our analysis revealed that 35 hormone-related genes were upregulated at least fourfold in *iqd1-1* plants, whereas 37 genes were down-regulated. While genes of the SA signaling pathway were mostly down-regulated in *iqd1-1* lines, ethylene-, ABA- and JA -responsive genes were noticeably up-regulated and genes involved in biosynthesis of these compounds were down-regulated. Three of the four down-regulated genes in the JA pathway are lipoxygenases (i.e., *LOX1*, *LOX5*, and *LOX6*) that function as JA-activated defense genes against biotic infection (Lõpez et al., 2011; Grebner et al., 2013; Viswanath et al., 2020). The fourth gene (*At1G09400*) encodes an NADPH dehydrogenase that participates in the JA biosynthesis pathway (Breithaupt et al., 2001). The most down-regulated hormone-related gene (*At3G21950*, 114.1-fold decreased expression, relative to WT) encodes a salicylic acid carboxyl methyltransferase, responsible for producing a volatile methyl ester that functions as a signaling molecule in the systemic defense against pathogens (Chen et al., 2003). Five of the eight upregulated ethylene pathway genes belong to the ERF/AP2 transcription factor family (i.e., *ERF9*, *ERF14*, *ERF15*, *ERF59*, and *ERF98*). These genes encode for ethylene response factor proteins that regulate the expression of defense responses genes following ethylene perception (Müller and Munné-Bosch, 2015). The ethylene biosynthesis genes *ACS2* and *ACO3* and the ethylene receptor-encoding *EIN4* gene were down-regulated in the *iqd1-1* mutant. We also observed that several genes linked to auxin and gibberellin, which are mainly related to growth and development, were also regulated in *iqd1-1 plants.* Some of these biosynthesis and metabolism genes were up-regulated whereas responsive genes demonstrated no specific trends in terms of expression (Table 1).

**TABLE 1:** Hormone-related genes differentially expressed in *iqd1-1* vs. WT plants (FC > 4).

Gene ID	Gene Description	Log_2_(FC)	Gene ID	Gene Description	Log_2_(FC)
**Auxin**			**Ethylene**		
AT1G51780	ILL5 (lAA-Leucine resistant Like 5)	3.243	AT5G20550	2OG-Fe(II)-Independent oxygenase	4.475
AT1G76190	SAUR56, Small Auxin Upregulated RNA	3.182	AT1G06160	ORA59 (Octadecanoid-Responsive Arabidopsis AP2/ERF 59)	2.536
AT3G07900	O-fucosyftransferase family protein	3.013	AT3G23230	ERF98 (Ethylene Response Factor 98)	2.354
AT2G18010	SAUR10	2.837	AT2G31230	ERF15 (Ethylene-responsive element binding factor 15)	2.326
AT5G55250	IAMT1 (IAA carboxyl methyltransferase)	2.749	AT1G04370	ERF 14 (Ethylene-responsive element binding factor 14)	2.235
AT4G34310	SAUR5	2.408	AT5G44210	ERF9 (ERF dDmain protein 9}	2.178
AT5G18060	SAUR23	2.256	AT5G67430	Acyl-CoA N-acyltransferase	2.106
AT5G18030	Auxin-responsive family protein	2.223	AT2G30830	2OG-dependent dioxygenase	2.086
AT4G34800	SAUR4	2.205	AT1G01480	ACS2 (ACC Synthase 2)	–2.038
AT5G18010	Auxin-responsive family protein	2.140	AT3G04580	EIN4 (Ethylene Insensitive 4)	–2.086
AT3G03830	Auxin-responsive family protein	2.112	AT5G09410	EICBP.B (Ethylene Induced Calmodulin Binding Protein)	–2.655
AT3G03340	SAUR27	2.063	AT5G5S530	2OG-dependent dioxygenase	–5.299
AT2G21220	SAUR12	2.013	AT1G12010	AC03 (ACC oxidase 3)	–6.656
AT1G60680	Aldo/keto reductase family protein	–2.075	**Cytokinin**		
AT5G20730	ARF7 (Auxin Response Factor 7)	–2.167	AT3G23630	IPT7 (Isopentenyltransferase 7)	2.128
AT3G54100	O-fucosyttransferase family protein	–2.177	AT5G35750	AHK2 (Arabidopsis Histidine Kinase 2)	–2.616
AT2G02560	CAND1 (cullin-Associated and Neddylation-Dissociated 1)	–2.204	AT2G01830	CRE1 (Cytokinin Response 1)	–2.95
AT1G60730	Aldo/keto reductase family protein	–2.320	AT2G17820	AHK1 (Arabidopsis Histidine Kinase 1)	–3.485
AT5G13320	PBS3 (AVRPPHB Susceptible 3)	–2.570	**Jasmonic Acid**		
AT2G34680	AIR9 (Auxin-Induced in Root Cultures 9)	–2.638	AT1G54040	ESP (Epithiospecifier protein)	6.444
AT5G09410	CAMTA1 (Calmodulin-Binding Transcription Activator 1)	–2.655	AT2G25980	Jacalin lectin family protein	3.153
AT1G28130	GH3.17 (IAA amido synthetase)	–2.749	AT5G42650	AOS (Allene Oxide Synthase)	2.081
AT4G27260	GH3.5 (IAA amido synthetase)	–2.985	AT3G22400	LOX5 (Lipoxygenase 5)	–2.215
AT5G54510	GH3 6 (IAA amido synthetase)	–2.986	AT1G09400	12-oxophytodienoate reductase	–2.366
AT5G55540	TRN1 (Tornado 1)	–3.084	AT1G67560	LOX6 (Lipoxygenase 6)	–2.631
AT2G23170	GH3 3 (IAA amido synthetase)	–3.096	AT1G55020	LOX1 (Lipoxygenase 1)	–3.703
AT3G02260	ASA1 (Attenuated Shade Avoidance 1)	–4.216	**Salicylic Acid**		
AT4G37390	GH3.2 (IAA amido synthetase)	–4.285	AT1G66690	SAM-dependent methyltransferase	2.235
**Abscisic Acid**			AT4G36470	SAM-dependent methyltransferase	–2.103
AT5G15960	KIN1 (cold and ABA inducible protein)	7.291	AT3G21950	SAM-dependent methyltransferase	–6.834
AT2G17770	BZIP27 transcription factor	4.182	**Gibberellin**		
AT1G75700	HVA22G (HVA22-like protein G)	2.861	AT3G46500	2OG-Fe(ll)-dependent oxygenase	3.967
AT3G02480	ABA-responsive protein-related	2.671	AT5G59845	Gibberellin-regulated family protein	3.182
AT2G47770	TSPO (Outer membrane Tryptophan- rich Sensory Protein-related)	2.485	AT5G37490	U-box domain-containing protein	2.774
AT2G27150	AA03 (Abscisic Aldehyde Oxidase 3)	–2.233	AT1G75750	GASA1 (GAST1 protein homolog 1)	2.354
AT1G16540	ABA3 (ABA Deficient 3)	–2.427	AT1G22690	Gibberellin-regulated family protein	2.155
AT3G43600	AA02 (Abscisic Aldehyde Oxidase 2)	–3.307	AT3G11540	SPY (Spindly)	–2.026
**Brassinosteroids**			AT4G25420	GA20OX1 (Gibberellin 20-Oxidase 1)	–2.309
AT3G20730	BIN3 (Brassinosteroid Insensitive 3)	–2.064	AT1G52320	2OG-Fe(ll)-dependent oxygenase	–2.565
AT1G74360	Leucine-rich repeat transmembrane protein kinase	–3.282	AT3G10185	Gibberellin-regulated family protein	–2.795

*Hormone biosynthesis or metabolism genes are in red and hormone response genes are in black. Data on gene annotation were obtained from the MapMan database.*

#### Activation of IQD1 by Hormones

**FIGURE 3:**
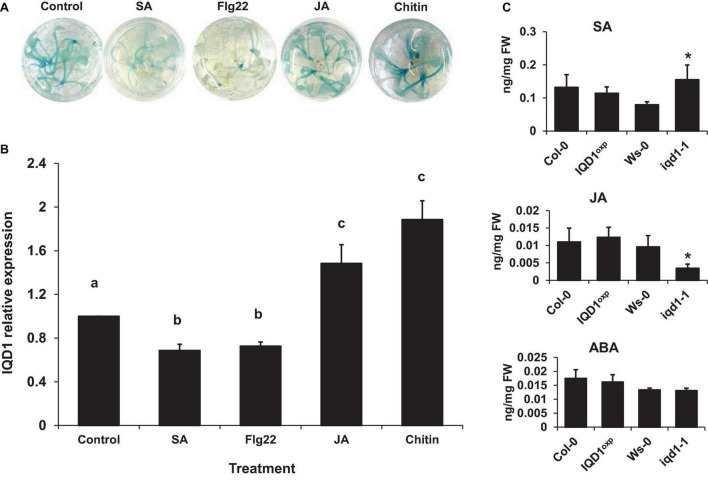
Elicitors affect IQD1 expression. Transgenic seedlings of gene trap line *IQD1pro:GUS* were treated with 100 μM salicylic acid, 100 nM Flg22, 100μM jasmonic acid, 500 μg/ml chitin or an equal volume of water as control for 18 h prior to histochemical GUS staining **(A)** or RNA extraction followed by qRT-PCR **(B)**. Results shown are from a biological replicate representative of six independent experiments for GUS staining and three for qRT-PCR. **(C)** SA, JA, and ABA accumulation in IQD1 mutants. Plant hormones were extracted from 3 week-old Arabidopsis seedlings grown on half-strength MS agar plates. Quantitative analysis of plant hormones was accomplished using LC-MS/MS with isotopically labeled analogs serving as internal standards. Each column represents an average of three independent biological replicates, with standard error bars indicated. Different letters above the columns indicate statistically significant differences at *P* < 0.05, as determined using Tukey’s honest significant difference test. Asterisks above the columns indicate statistically significant differences relative to WT plants at *P* < 0.05, as determined using Student’s *t*-test.

We observed that a large number of genes responsible for defense hormone response were altered in the *iqd1-1* line, as compared to the WT, according to the RNA-Seq results. This prompted us to investigate the effects of exogenous hormone and elicitor treatments on IQD1 expression in Arabidopsis seedlings. To this end, we used the β-glucuronidase (GUS) reporter line, *IQD1*^pro^*:GUS*, (*iqd1-2*, GT6935 line) that contains a fusion of the IQD1 promoter and a β-glucuronidase enzyme (Sundaresan et al., 1995). Histochemical staining of the reporter plants following treatment with SA or Flg22, a known activator of the SA signal transduction, showed marked downregulation of *IQD1* expression as evident by decreased GUS staining (Figure 3A) and by qRT-PCR analysis (Figure 3B). In contrast, application of free JA or chitin, a major component of fungal cell walls, led to activation of *IQD1* expression, further confirming the link between *IQD1* activity and the JA pathway (Figures 3A,B).

We also extracted plant hormones from *iqd1-1* mutant plants and noted significantly lower JA levels, as compared to WT *A. thaliana*. We also observed significantly increased SA levels but no difference in ABA levels. At the same time, there were no changes in the JA, SA, or ABA content of *IQD1*^OXP^** plants (Figure 3C). These results suggest a role for *IQD1* in JA accumulation and/or a synergistic effect between JA and SA signaling.

#### Dissection of IQD1 Integration Into Defense Hormone Pathways

**FIGURE 4:**
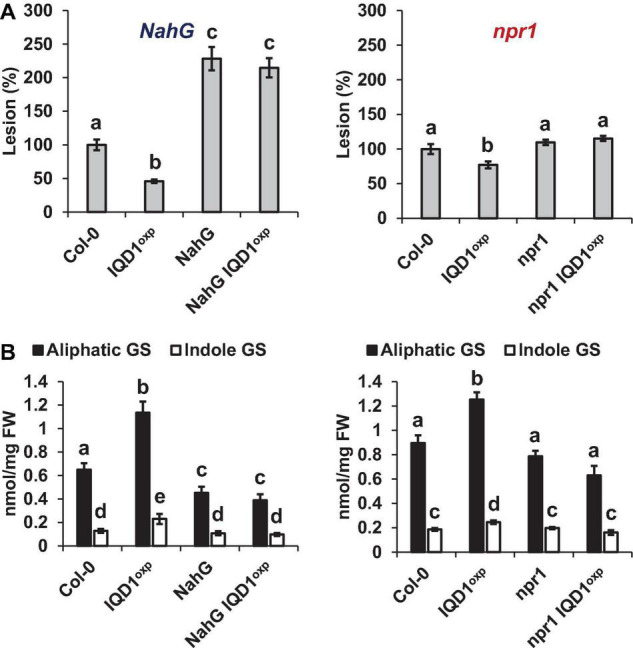
*IQD1*^OXP^** affects SA pathway mutants. **(A)** Detached leaves from 6 week-old *Arabidopsis* SA pathway mutants were inoculated with *B. cinerea*. Lesion sizes were measured 72 h post-inoculation. Average lesion sizes from 30 leaves of each line are presented, along with the standard error of each average. All numbers are presented as the relative percentage to the corresponding background wild-type. Different letters above the columns indicate statistically significant differences at *P* < 0.05, as determined using the Tukey’s honest significant difference test. **(B)** Glucosinolates were extracted from 6-week old *Arabidopsis* seedlings of SA pathway mutants and analyzed by HPLC. Mean contents of methionine-derived (black bars) and tryptophan-derived (gray bars) glucosinolates are given for each line. Each column represents an average of eight seedlings, with standard error bars indicated. Different letters above the columns indicate statistically significant differences at *P* < 0.05, as determined using Tukey’s honest significant difference test. Results shown are from a biological replicate representative of three independent experiments.

To investigate IQD1 integration into the biosynthesis and response pathways of the three defense hormones, we tested the relationships between *IQD1* and *A. thaliana* hormone-related mutants. We constructed homozygous double mutants by crossing the enhancer trap *IQD1*^OXP^** line with mutants defective in plant-hormone synthesis and signal response. The *NahG* line, a transgenic line expressing a bacterial salicylate hydroxylase that converts SA into catechol, leading to a dramatic decrease in plant SA content, showed increased sensitivity to *B. cinerea* as compared to WT and *IQD1*^OXP^**, a phenotype that was not abolished in *NahG IQD1*^OXP^** double transgenic plants (Figure 4A). We also determined GS concentrations in the double transgenic plants and found that aliphatic GS content is reduced in both the single and double *NahG* transgenic lines, as compared to WT and *IQD1*^OXP^** plants (Figure 4B). We observed no difference in disease severity or GS accumulation in the SA regulator *npr1* mutant line or the *npr1 IQD1*^OXP^** cross (Figures 4A,B).

**FIGURE 5:**
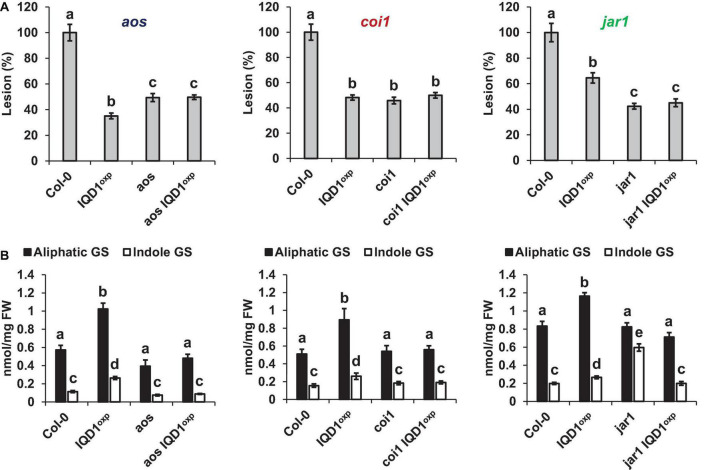
*IQD1*^OXP^** affects JA pathway mutants. **(A)** Detached leaves from 6 week-old *Arabidopsis* JA pathway mutants were inoculated with *B. cinerea*. Lesion sizes were measured 72 h post-inoculation. Average lesion sizes from 30 leaves of each line are presented along with the standard error of each average. All numbers are presented as the relative percentage to their corresponding background wild-type. Different letters above the columns indicate statistically significant differences at *P* < 0.05, as determined using Tukey’s honest significant difference test. **(B)** Glucosinolates were extracted from seedlings of 6 week-old *Arabidopsis* SA pathway mutants and analyzed by HPLC. Mean contents of methionine-derived (black bars) and tryptophan-derived (gray bars) glucosinolates are given for each line. Each column represents an average of eight seedlings, with standard error bars indicated. Different letters above the columns indicate statistically significant differences at *P* < 0.05, as determined using Tukey’s honest significant difference test. Results shown are from a biological replicate, representative of three independent experiments.

All three JA pathway-related mutant lines (i.e., *aos*, *coi1*, and *jar1*) and their crosses with *IQD1*^OXP^** were more resistant to *B. cinerea* infection than were WT plants. While the *aos* and *aos IQD1*^OXP^** mutants exhibited an intermediate resistance, falling between those presented by *IQD1*^OXP^** and WT plants, the responses of *coi1* and *coi1 IQD1*^OXP^** lines were undistinguishable from *IQD1*^OXP^**plants. The *jar1* line and the *jar1 IQD1*^OXP^** crossed line displayed exceptionally high resistance to *B. cinerea*, surpassing even that of *IQD1*^OXP^** (Figure 5A). However, while GS content in the *aos IQD1*^OXP^** and *coi1 IQD1*^OXP^** lines remained unchanged, as compared to the parental lines, the *jar1 IQD1*^OXP^** plants displayed altered GS content. Indole GS content in the *jar1* plants was higher even than that of the *IQD1*^OXP^** line. Indole GS concentrations in the *jar1 IQD1*^OXP^** plants were lower than what was seen in the *jar1* parent plants and were comparable to WT levels (Figure 5B).

**FIGURE 6:**
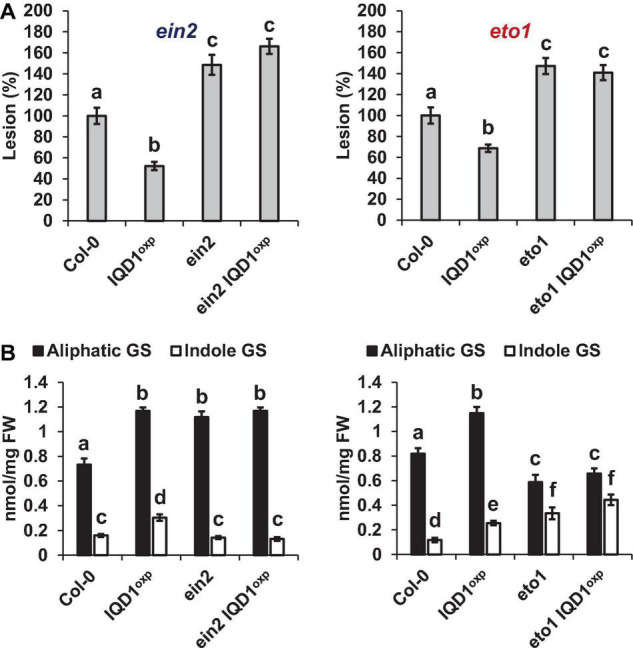
*IQD1*^OXP^** effect on ethylene pathway mutants. **(A)** Detached leaves from 6 week-old *Arabidopsis* ethylene pathway mutants were inoculated with *B. cinerea*. Lesion sizes were measured 72 h post-inoculation. Average lesion sizes from 30 leaves of each line are presented along with the standard error of each average. All numbers are presented as the relative percentage to their corresponding background wild-type. Different letters above the columns indicate statistically significant differences at *P* < 0.05, as determined using Tukey’s honest significant difference test. **(B)** Glucosinolates were extracted from 6-week old *Arabidopsis* seedlings of SA pathway mutants and analyzed by HPLC. Mean contents of methionine-derived (black bars) and tryptophan-derived (gray bars) glucosinolates are given for each line. Each column represents an average of eight seedlings with standard error bars indicated. Different letters above the columns indicate statistically significant differences at *P* < 0.05, as determined using Tukey’s honest significant difference test. Results shown are from a biological replicate, representative of three independent experiments.

As demonstrated in Figure 6A, both the Arabidopsis ethylene over-producing *eto1* line and the ethylene signaling pathway *ein2* mutant were more sensitive to *B. cinerea* than were WT and *IQD1*^OXP^** plants. Siblings of *ein2 IQD1*^OXP^** and *eto1 IQD1*^OXP^** plants failed to rescue this phenotype. Aliphatic GS levels in *eto1* and *eto1 IQD1*^OXP^** plants were lower than in *IQD1*^OXP^** and WT plants, while indole GS levels were higher. Although indole GS levels in *eto1* plants and the crossed line *eto1 IQD1*^OXP^** were higher even than in *IQD1*^OXP^** plants, this was not reflected in the resistance of those lines to *B. cinerea* infection (Figure 6B), probably due to the lower levels of aliphatic GSs. Indole GS levels in the *ein2* and *ein2 IQD1*^OXP^** lines were lower than in *IQD1*^OXP^** plants and comparable to what was seen in the WT, while aliphatic GS levels were higher than in WT and comparable to what was measured in the *IQD1*^OXP^** line (Figure 6B).

#### Involvement of IQD1 in Glucosinolate Biosynthesis

RNA-Seq transcriptional analysis of *iqd1-1* lines, as compared to WT plants, revealed altered expression of GS-related genes. Our analysis shows that out of seven DEGs, six were downregulated in the mutant and only one was upregulated (Supplementary Figure 4 and Supplementary Data Sheet 1). Among the genes that were downregulated was *MAM1* that encodes a methylthioalkylmalate synthase, which catalyzes the condensation reactions of the first two rounds of methionine chain elongation in the biosynthesis of methionine-derived glucosinolates (Textor et al., 2004). *FMO GS-OX2* encodes a glucosinolate S-oxygenase that catalyzes the conversion of methylthioalkyl glucosinolates to methylsulfinylalkyl glucosinolates (Li et al., 2008). *CYP79B2*, which belongs to the cytochrome P450 gene family, is involved in tryptophan metabolism and indole GS biosynthesis (Mikkelsen et al., 2000). *TGG2* is a myrosinase-encoding gene involved in catabolizing GSs into active products (Barth and Jander, 2006). *GLL23* encodes a myrosinase-associated protein belonging to a large plant GDSL-like lipase family (Jancowski et al., 2014). *ESM1* represses nitrile formation and favors isothiocyanate production during glucosinolate hydrolysis (Zhang et al., 2006). The only up-regulated GS related gene in *iqd1-1* plants was *ESP*, encoding an epithiospecifier protein that promotes the creation of nitriles instead of isothiocyanates during glucosinolate hydrolysis (Lambrix et al., 2001). Levels of MYB transcription factors involved in GS accumulation were mostly not changed in *iqd1-1* vs. WT plants, although *MYB122*, involved in indole GS control, was down-regulated (Frerigmann and Gigolashvili, 2014a; Table 2). Other MYB genes that control GS synthesis, such as *MYB28*, *MYB29*, and *MYB34* were also down-regulated in the *iqd1-1* line but only after infection with *B. cinerea* (Supplementary Data Sheet 4). These results corroborate the active role of IQD1 at different steps of GS biosynthesis, as seen earlier with loss- and gain-of-function *A. thaliana* lines (Levy et al., 2005).

**TABLE 2:** Differentially expressed genes involved in GS biosynthesis, regulation, and hydrolysis in the *iqd1-1* line, as compared to WT plants.

*#*	Gene name	Description	Log_2_(fold change)
**1**	ESP	Epithiospecifier	6.44
**2**	CYP79B2	Tryptophan metabolism	−2.14
**3**	MAM1	Methylthioalkylmalate synthase	−2.18
**4**	FMO GS-OX2	GS S-oxygenase	−2.87
**5**	GLL23	Myrosinase associated protein	−2.94
**6**	ESM1	Represses nitrile formation	−6.10
**7**	TGG2	Myrosinase	−8.92
**8**	MYB122	Transcription factor	−3.55

### Involvement of IQD1 in *Botrytis cinerea* Pathogenicity

In this study, we also analyzed the gene expression profiles of *B. cinerea* infecting the *IQD1* knockout line (*iqd1-1* mutant), as compared to infecting WT plants (for statistical analysis of the raw data for each sample after sequencing, see Supplementary Table 2).

#### Identification of *Botrytis cinerea* Differentially Expressed Genes Following Wild Type and *iqd1-1* Plant Infection

Unique reads that perfectly matched reference genes in each library (i.e., *B. cinerea* infecting WT or *iqd1-1* plants) were used to generate a matrix of normalized counts and perform statistical tests to determine whether genes were differentially expressed between pairs of factor combinations. *B. cinerea* genes with less than fourfold differences in either infecting WT or infecting *iqd1-1* plants were excluded from further analyses (Supplementary Data Sheet 6). The frequencies of genes with different fold changes in expression is shown in Supplementary Figure 5A. A total of 678 *B. cinerea* genes were differentially expressed when infecting the *iqd1-1* mutant, as compared to the WT (fold change > 4). These included 466 up-regulated genes (i.e., genes expressed at higher levels when infecting the *iqd1-1* mutant, represented as positive values on the *Y*-axis in Supplementary Figure 5A) and 212 down-regulated genes (i.e., genes expressed at higher levels when infecting the WT, represented as negative values on the *Y*-axis in Supplementary Figure 5A). We found that 84% of the up-regulated DEGs (391 genes) showed fold changes in the 4–20 range, while levels of the remaining 16% (75 genes) changed from a 20-fold and to a near 4,000-fold difference. At the same time, 92% (194 genes) of the downregulated DEGs showed a fold change difference lower than 10, while the levels of only 8% (18 genes) changed more than tenfold.

To validate the RNA-Seq data, six genes were selected for qRT-PCR analysis, namely, *Bc1G_11623* (encoding a MFS sugar transporter), *Bc1G_10358* (encoding a hypothetical protein), *Bc1G_04691* (encoding a cellulase), *Bc1G_02144* (encoding a choline dehydrogenase), *Bc1G_12885* (encoding a MFS transporter) and *Bc1G_13938* (encoding a sialidase). The expression patterns of these genes obtained by qRT-PCR and RNA-Seq were similar, indicating that the results from the RNA-Seq data are indeed indicative of the *B. cinerea* transcriptome (Supplementary Figure 5B).

#### Functional Annotation of *Botrytis cinerea* Differentially Expressed Genes After Infection of *iqd1-1* Mutant

**FIGURE 7:**
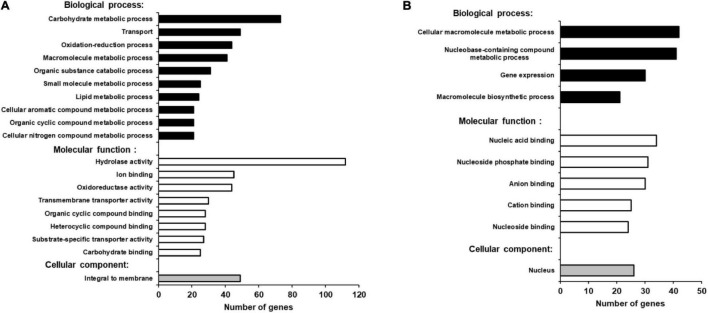
GO enrichment analysis of up-regulated *B. cinerea* genes. Significantly enriched GO terms classified by biological process, molecular function and cellular component when infecting *iqd1-1* **(A)** or WT plants **(B)**. Only GO terms that applied to more than 20 differentially expressed genes are shown.

Blast2Go bioinformatics software was used to identify gene functions in the annotated *B. cinerea* genome, where more than 85% of the genes have yet to be assigned a function (Staats and van Kan, 2012). Based on the overall analysis of gene expression profiles presented here, we were able to find BLAST hits to 460 up-regulated genes (98.7%) and GO (Gene Ontology) annotations for 268 genes (57.5%) from *B. cinerea* infecting *iqd1-1* plants. The proteins encoded by the DEGs are mainly located in the plasma membrane, when classified by cellular components (Figure 7A). When classified according to biological processes and molecular function these proteins were assigned hydrolase, oxidoreductase and trans-membrane transporter activities and they were identified as participating in carbohydrate catabolism, oxidation-reduction processes and molecule transport across the plasma membrane (Figure 7A). As stated above, only 212 *B. cinerea* genes displayed higher expression levels when infecting WT plants, relative to when infecting the *iqd1-1* mutant. Moreover, differences in expression of these genes amounted to less than 20-fold, at most. Using Blast2Go software, we managed to find BLAST hits for 204 DEGs (96.2%) and GO annotations for 115 genes (54.2%). The proteins encoded by the DEGs show a propensity for nuclear localization, when classified for cellular component. Their predicted molecular functions included nucleic acid binding, helicase and kinesin activities and participation in macromolecule and nucleobase biological metabolic processes and gene expression (Figure 7B).

#### Highly Expressed *Botrytis cinerea* Genes After Infection of *iqd1-1* Plants

To further elucidate the specific functions of their DEGs, those *B. cinerea* genes showing a more than 50-fold change in expression upon infecting *iqd1-1* plants were further analyzed. This group comprised the top 30 up-regulated *B. cinerea* genes when infecting the *iqd1-1* line (Table 3). The most abundant group of proteins are involved in the degradation of complex carbohydrates and listed as Carbohydrate-Active-Enzymes (CAZymes) (Garron and Henrissat, 2019). In fact, 20 of the 30 genes (67%) on this list are CAZymes that participate in the breaking down of the host plant’s primary and secondary cell walls. Specifically, these genes encode enzymes such as cellulases, hemicellulases, pectinases and other related proteins. Seven of the DEGs (23%) encode products belonging to the Major Facilitator Superfamily (MFS) and exhibited more than 50-fold change in expression. The MFS comprises a class of membrane proteins that facilitate the transport of small solutes, such as sugars and antibiotics, across the cell membrane (Yan, 2015; Niño-González et al., 2019). The remaining three genes in the list encode for a fungal extracellular membrane protein with an anticipated role in pathogenesis, a transmembrane protein with proposed glucose transport activity and a hypothetical protein of unknown function.

**TABLE 3:** *D*ifferentially expressed *B. cinerea* genes showing more than 50-fold changes in their expression when infecting *iqd1-1*, as compared to WT plants.

Gene annotation	Log_2_(FC)	Gene annotation	Log_2_(FC)
Cellulase	11.96	Hemicellulase	6.49
Extracellular membrane protein	11.57	MFS sugar transporter	6.43
Cellulase	11.28	MFS sugar transporter	6.32
Cellulase	10.46	MFS sugar transporter	6.26
Hemicellulase	9.06	Cellulase	6.1
Cellulase	9.06	Hemicellulase	6.09
Cellulase	7.78	Hypothetical protein	6.04
Transmembrane protein	7.57	Celllulosome complex protein	5.95
Cellulase	7.56	Pectinase	5.94
Hemicellulase	7.13	MFS transporter	5.82
Cellulase	7.12	Cellulase	5.79
MFS sugar transporter	7.12	Pectinase	5.74
MFS sugar transporter	7.06	Hypothetical protein	5.71
Cellulase	6.8	Cellulase	5.68
MFS sugar transporter	6.7	Pectinase	5.67

#### CAZyme Distribution in Differentially Expressed Genes

**FIGURE 8:**
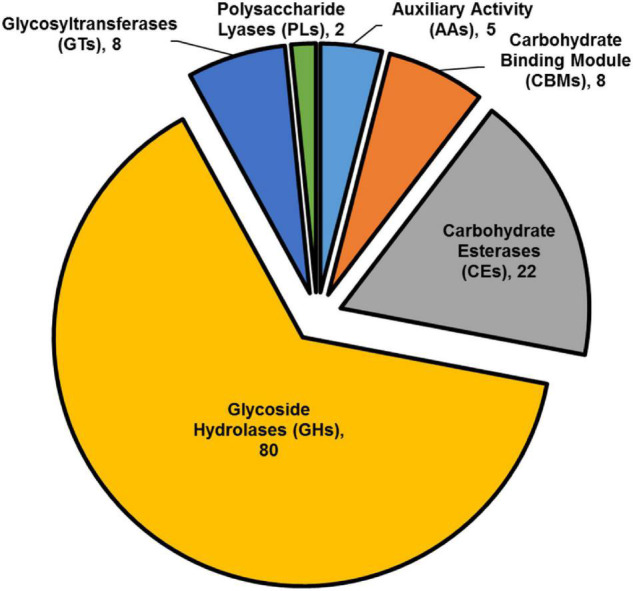
Classification of CAZymes encoded by up-regulated DEGs. The contribution of each CAZymes family is shown. Numbers in brackets denote the number of DEGs for each family.

The striking number of CAZymes encoded by members of the highly differentially expressed gene list, prompted us to investigate their distribution among the upregulated DEGs. We found that CAZymes comprise 125 of 466 genes (27%) that were up-regulated in *B. cinerea* infecting *iqd1-1* plants, while only 18 of 212 genes (8%) were upregulated in *B. cinerea* that infect WT plants. It was surprising to see that *in B. cinerea* inoculating WT Arabidopsis plant only 8% CAZymes-encoding genes were up-regulated, although it should be noted that we compared *B. cinerea* inoculating *iqd1-1* and WT plants and not *B. cinerea* grown on culture medium. The largest group (80 genes corresponding to 64%) encode CAZymes belonging to the glycoside hydrolase family that constitute lytic enzymes, such as cellulases and hemicellulases. The second largest group (22 genes, 18%) encode carbohydrate esterases that incorporate pectin catabolic enzymes. The remaining CAZymes operate on other constituents of the plant cell wall or play auxiliary roles to other enzymes (Figure 8).

## Discussion

This study aimed to elucidate the molecular functions of the *A. thaliana* IQD1 protein in defense responses against the plant pathogen *B. cinerea*. Previous work with IQD1 mutants showed that the *IQD1* expression levels in different *A. thaliana* lines correlated with steady state accumulation of glucosinolates. Moreover, these earlier efforts showed that overexpressing *IQD1* has the beneficial characteristic of reducing the herbivory of generalist insects (Levy et al., 2005). Using the necrotrophic fungal pathogen *B. cinerea*, we sought to investigate the cellular and genetic pathways that IQD1 regulated and thus affects plant defense response. Inoculating the *IQD1*^OXP^** and *iqd1-1* lines with a *B. cinerea* spore suspension proved the correlation between *IQD1* expression levels and *A. thaliana* resistance to the fungal pathogen (Figure 1), as well as providing us with a simple host-pathogen system to conduct genetic screening. It was already known from our previous studies that the *iqd1-1* knockout plant accumulates low levels of GS (Levy et al., 2005). In the current study, we also validated that *iqd1-1* plants abnormally express several GS biosynthesis and regulation genes, as compared to WT plants (Table 2).

Information obtained from genome-wide expression profiling of *iqd1-1* and WT plants following mock treatment or *B. cinerea* infection, helped us understand which plant metabolic processes were affected by the absence of IQD1. The latest genome model released for *A. thaliana* (TAIR10) contains about 27,000 protein-coding genes (Lamesch et al., 2012). We showed that approximately 3,500 genes (roughly 13% of all coding genes) were differentially expressed in the non-infected IQD1 knockout as opposed to WT plants (Supplementary Figure 1A and Supplementary Data Sheet 1). Furthermore, 70% of the genes which were downregulated in the *iqd1-1* line generate products predicted to serve diverse functions, including transport, DNA repair and gene regulation. It is noteworthy that a large number of downregulated genes in the *iqd1-1* line encode proteins responsible for plant defense against biotic stresses, such as cell wall remodeling proteins, signaling factors and proteins involved in resistance (Figure 2). Such a massive impairment of the plant defense apparatus is likely to explain the enhanced sensitivity of the knockout plants to insect and pathogen attacks (Levy et al., 2005; Figure 1). The ERF genes encode a large family of ethylene responsive transcription factors that regulate important biological processes related to plant growth, development and plant defense (Nakano et al., 2006; Li et al., 2019). This gene family was largely upregulated in the *iqd1-1* mutant (Figure 2 and Table 1). The increased sensitivity to ethylene may explain several phenotypes displayed by this line, such as rapid growth and early development of stems and seed pods, relative to WT plants (Levy et al., 2005). As demonstrated in Figure 6 and earlier studies, ethylene can effect glucosinolate biosynthesis (Mikkelsen et al., 2003) and its signaling components EIN2 and ETO1 act downstream to IQD1 control of defense and GS accumulation.

Upon inoculation with *B. cinerea*, both the WT (Supplementary Figure 2) and *iqd1-1* (Supplementary Figure 3) plants presented a similar basic transcriptional response, shutting down the energy-consuming photosynthesis machinery and instead concentrating efforts on fighting off the invading pathogen. The responses did, however, differ with the WT plants being able to express more defense-related genes, like those encoding germins and R-genes (Figure 2B), thus more effectively resisting fungal infection than *iqd1-1* plants. As for other IQD family members, our transcriptional data demonstrated that while most genes were unaffected after *B. cinerea* infection, three were up-regulated in response to *B. cinerea* inoculation (i.e., IQD13, IQD15, and IQD27) and five are down-regulated (i.e., IQD9, IQD18, IQD19, IQD21, and IQD26). Since IQD genes products serve a wide range of cellular functions, many unrelated to defense mechanisms, it is not surprising that the levels of these genes differed as a function of the biological stress imposed in the current study.

**FIGURE 9:**
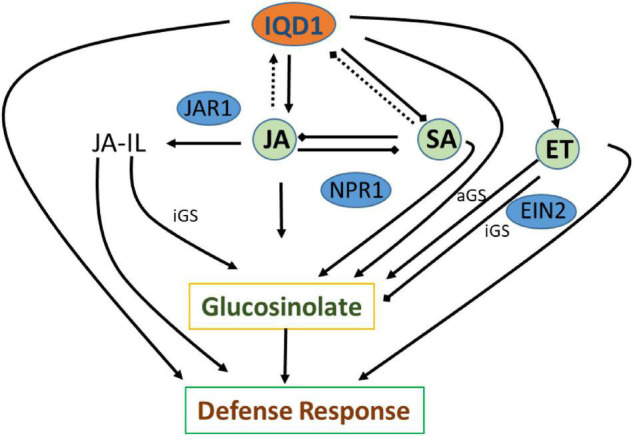
Suggested model of IQD1 involvement in glucosinolate accumulation and defense responses. Intact arrows indicate positive connections (→), intact lines with diamond heads indicate negative connections (→), and dashed lines indicate effects on expression (…..). JA-Il, jasmonic acid isoleucine; aGS, aliphatic glucosinolate; iGS, indolic glucosinolate; JAR1, JASMONATE RESISTANT 1; NPR1, NON-EXPRESSER OF PR GENES1; EIN2, ETHYLENE INSENSITVE 2.

The three plant hormones SA, JA, and ethylene play a major role in response to biotic stresses by mediating endogenous signaling that activates the expression of plant defense genes (Dong, 1998; Clarke et al., 2000; Li et al., 2019). Analysis of RNA-Seq data of *iqd1-1* plants indicated that IQD1 is involved in all three major hormone defense pathways (Table 1). While we saw transcriptional changes in genes controlling all important plant hormones in WT and mutant plants, ethylene JA and ABA signaling genes were mainly upregulated in *iqd1-1* plants (see above), unlike to SA metabolism genes that showed opposite behavior (Table 1). Using the *IQD1*^pro^*:GUS* reporter line we showed that exogenous application of SA or Flg22 down-regulates *IQD1* expression, while JA and chitin treatment led to the opposite effect, activating *IQD1* expression (Figures 3A,B). Further confirmation of the link between IQD1 activity and the JA pathway came from LC/MS quantification of hormone accumulation in IQD1 mutants. We observed lower steady-state JA levels in *iqd1-1* mutant plants, as compared to WT, while SA levels were significantly increased (Figure 3C). We can speculate that IQD1 represses the accumulation of SA while activating JA accumulation (Figures 3C, 9 and Table 1). It is clear from former publications that glucosinolate accumulation and metabolism are under the control of different hormone signaling pathways and several studies have demonstrated, like us, that changes in glucosinolate levels altered levels of hormone, such as JA, ET, and ABA (Mikkelsen et al., 2003; Dombrecht et al., 2007; Malitsky et al., 2008; Morant et al., 2010; Chen et al., 2011; Mitreiter and Gigolashvili, 2021). Given the results on hormone levels in the *iqd1-1* mutant and expression levels of *IQD1* after hormone treatment presented above, we hypothesize that the opposing effects on SA and JA levels reflected the involvement of IQD1 in the well documented synergy between the JA and SA pathways (Koornneef and Pieterse, 2008; Pieterse et al., 2012; Li et al., 2019; Figure 9).

GS metabolism is also linked to auxin homeostasis. Indole GS contribute to auxin biosynthesis via metabolic intermediates indole-3-acetaldoxime (IAOx) and indole-3-acetonitrile (IAN). IAOx thus constitutes a metabolic branch point in indole-3-acetic acid (IAA) and indole glucosinolate biosynthesis, while IAA levels can be regulated by the flux of IAOx (Bak et al., 2001; Malka and Cheng, 2017). We observed some up-regulation of auxin responsive genes in our RNA-seq study on the one hand, yet also saw the same number of down-regulated genes. On the other hand, we did not observe any auxin-related phenotype in the *iqd1-1* or *IQD1*^OXP^** lines, leading us to assume that auxin levels might not change, as also seen in the of *CYP79B2* overexpressing line, in which significantly elevated levels of indole GSs and IAN but normal IAA levels were seen (Mikkelsen et al., 2000; Zhao et al., 2002; Glawischnig et al., 2004). These observations raise the possibility that the IAOx pathway may not contribute to basal IAA production and to its role in regulating plant growth and development as much as other IAA biosynthesis pathways. There is some evidences that other IQD family members, such as IQD15, are connected to the auxin response (Möller et al., 2017), although *IQD1* expression levels were not affected after auxin treatment in various transcriptional studies (Pufky et al., 2003; Nemhauser et al., 2004; Redman et al., 2004). Clearly, the connection between IQD1 and auxin needs to be further characterized. Based on data obtained following *B. cinerea* inoculation of detached leaves and GS concentration measurements by HPLC on siblings of *IQD1*^OXP^** crossed with different transgenic plants and mutants connected to hormonal pathways, we were able to investigate IQD1 integration into the three main defense hormone-signaling and response pathways. Over-expression of *IQD1* did not alter resistance/sensitivity or GS levels in any of SA and ethylene pathway mutants we tested (Figures 4, 6). We thus, assume it to be more likely that IQD1 is dependent on them and that it might act upstream to hormone pathways for defense activation and GS accumulation (Figure 9). The data presented here showed that the *ein2* and *eto1* mutants were both more sensitive to *B. cinerea* from WT plants despite presenting opposite phenotypes in terms of ethylene responses. This discrepancy can be explained by the difference in GS levels: in the *ein2* mutant, levels of indole GS are reduced and levels of aliphatic GS are increased, whereas the reverse was found in the *eto1* mutant where levels of indole GS are increased and levels of aliphatic GS are reduced (Figure 6B). This perhaps reflects that GSs having a greater impact than ethylene on this *B. cinerea* isolate (Buxdorf et al., 2013). Additionally, ethylene has also been shown to induce the expression of GS biosynthetic genes and their regulators (Mikkelsen et al., 2003; Frerigmann and Gigolashvili, 2014b). Another possibility is that the elevated levels of ethylene induced *B. cinerea* pathogenicity (Elad, 1990; Chagué et al., 2006). Furthermore, while indole GS content in the *jar1* plants was higher even than in the *IQD1*^OXP^** line, most likely due to the increase of several JA conjugates in the single mutant, as described before (Staswick and Tiryaki, 2004), the *jar1 IQD1*^OXP^** cross plants accumulated significantly less indole GSs than did the *jar1* plants but similar to what was seen in WT plants (Figure 5). These results offer additional proof of the connection between IQD1 and the JA pathway. We thus, hypothesize that IQD1 acts upstream of the JA signaling pathway and is dependent on JAR1 controlled indole GS accumulation. IQD1 also controls JA accumulation by activating JA biosynthesis genes (Table 1) and is activated by JA via a positive feedback loop (for model, see Figure 9).

The extensive volume of data obtained from our RNA-Seq experiment also enabled us to investigate the properties of *B. cinerea* infection on *iqd1-1* plants, as compared to the WT (Figure 7A). Examination of differentially expressed genes (DEGs) revealed that upon *iqd1-1* infection, the fungus expressed an extensive array of CAZymes and membrane transporters, which facilitate the penetration and breakdown of plant tissues (Table 3 and Figure 8). It has been proposed that *B. cinerea* is able to fine tune the expression of activated CAZymes according to the carbohydrate composition of the host cell wall (Blanco-Ulate et al., 2014).

We thus hypothesize that following early penetration of the leaf tissue, the fungus can better proliferate on *iqd1-1* plants since this mutant contains low levels of GSs and this can induce the expression of CAZymes that rapidly break down the physical barriers of the plant cell (Table 3 and Supplementary Figure 6). We conclude that *B. cinerea* infection is more aggressive on *iqd1-*1 plants, as the fungus takes advantage of the enhanced sensitivity of the mutant, mainly the reduction in GS levels, as also described in our previous work (Buxdorf et al., 2013).

In conclusion, the current study demonstrated that altered expression of *A. thaliana* IDQ1 has a profound effect on the global expression of plant genes but also those of the pathogen. Moreover, *IQD1* expression correlates with GS levels, defense signaling and *B. cinerea* pathogenicity.

## Experimental Procedures

### Plant Lines and Growth Conditions

This work was carried out using the following *A. thaliana* (L.) Heynh. background lines: Columbia (*Col-0*) and Wassilewskija (*Ws-0*). The following mutants and transgenic plants were used in *Col-0* background: *IQD1*^OXP^** an enhancer trap line, which contains four repeats of the enhancer region of the constitutively active 35S promoter of *cauliflower mosaic virus* adjacent to *IQD1* gene (Levy et al., 2005), *NahG* (Delaney et al., 1994), *npr1-1* (Cao et al., 1994), *aos* (Park et al., 2002), *coi1* (Xie et al., 1998), *jar1-1* (Staswick et al., 1992), *ein2-1*, and *eto1-1* (Guzmán and Ecker, 1990). In *Ler* background: *iqd1-2* gene trap line GT6935 (Levy et al., 2005). In *Ws-0* background: T-DNA insertion line *iqd1-1* (Levy et al., 2005). All seeds were stratified on moist soil at 4°C for 2–3 days before placing them in a growth chamber. Arabidopsis plants were grown at 22°C and 60% relative humidity under illumination with fluorescent and incandescent light at a photofluency rate of approximately 120 μmol m^–2^ s^–1^, day length was 10 h unless otherwise specified.

To obtain double mutants, each individual mutant was crossed with the *IQD1*^OXP^** line. F1 populations were screened on Basta herbicide introduced in the *IQD1*^OXP^** line (glufosinate ammonium). Double homozygous mutants were identified in the F2 populations by PCR analysis using the allele-specific primer pairs listed in Supplementary Table 3. PCR analysis only yields an amplified product if an untransformed wild-type allele exists (such as in heterozygous plants). In homozygous plants the binding sites for the primers are interrupted in both alleles by the T-DNA insertion, thus yielding no PCR product. These plants were self-crossed and further progeny from a homozygous line was used for experiments. Double mutants with NahG transgene were conferred as homozygous only when the transgene PCR product was detected in the entire siblings of self-progeny.

### Fungal Strains, Growth, and Inoculation Method

*Botrytis cinerea* (GRAPE isolate, isolated in 2001 from an infected grapevine leaf from a vineyard in northern California) was grown on potato dextrose agar (PDA; Difco, Le Pont de Claix, France) in a controlled-environment chamber kept at 22°C under fluorescent and incandescent light at a photofluency rate of approximately 120 μmol m^–2^ s^–1^ and a 10/14 h photoperiod.

Conidia were harvested in sterile distilled water and filtered through a 45 μm cell strainer to remove hyphae. For inoculation, the conidial suspension was adjusted to 1,500 conidia/μl in half-strength filtered (0.45 μm) grape juice (pure organic). Leaves were inoculated with 4 μl droplets of conidial suspension prior to RNA purification. Detached leaves from the different genotypes were layered on trays of water-agar media and inoculated with 4 μl droplets of conidial suspension. Lesions were measured using ASSESS 2.0, image analysis software for plant disease quantification (APS Press, St. Paul, MN, United States).

### β-Glucuronidase Histochemical Assay

To carry out GUS reporter gene staining assays, *iqd1-2* (GT6935 line) seeds were sterilized in (70% ethanol, 0.05% tween 20) for 5 min, washed with 100% ethanol and left to air dry. Seeds were germinated in 12-well microtiter dishes sealed with parafilm, each well containing 3 seeds and 2 ml seedling growth medium [SGM; 0.5× Murashige and Skoog basal medium with vitamins (Duchefa, Haarlem, The Netherlands) containing 0.5 g/L MES hydrate and 1% sucrose at pH 5.7]. Seedlings were grown for 14 days in a growth chamber with continuous shaking at 100 rpm before treatment with elicitors. Elicitors were used at the following concentrations: 100 μM SA, 100 μM JA, 100 nM Flg22, and 500 μg/ml chitin. 18 h after treatment with elicitors, seedlings were either taken for RNA isolation (see below) or 2 ml of GUS substrate solution [125 mM sodium phosphate pH 7, 1.25 mM EDTA, 1.25 mM K_4_[Fe(CN)_6_], 1.25 mM K_3_[Fe(CN)_6_], 0.5 mM X-Gluc and 1.25% Triton X-100] was poured in each well. The plants were vacuum-infiltrated for 10 min and then incubated at 37°C overnight covered in aluminum foil. Tissues were de-stained with 100% ethanol overnight and placed in 70% ethanol before digital pictures were taken.

### LC/MS Quantification of Salicylic, Jasmonic, and Abscisic Acid

Quantitative analysis of plant hormones was accomplished using LC-MS/MS system which consisted of a 1,200 series Rapid Resolution liquid chromatography system (vacuum micro degasser G1379B, binary pump G1312B, autosampler G1367C and thermal column compartment G1316B) coupled to 6,410 triple quadruple mass selective detector (Agilent Technologies, Santa Clara, CA, United States). Analytes were separated on an Acclaim C18 RSLC column (2.1 × 150 mm, particle size 2.2 μm, Dionex) upon HPLC conditions described in Supplementary Table 4.

Mass spectrometer was operated in negative ionization mode, ion source parameters were as follows: capillary voltage 3500V, drying gas (nitrogen) temperature and flow 350°C and 10 l/min, respectively, nebulizer pressure 35 psi, nitrogen (99.999%) was used as a collision gas. The LC-MS system was controlled and data were analyzed using MassHunter software (Agilent Technologies). Quantitative analysis of plant hormones was accomplished in multiple reaction monitoring (MRM) mode, isotopically labeled analogs were used as internal standards. MRM parameters are listed in Supplementary Table 5.

### Glucosinolate Extraction and Purification

Six weeks old soil grown *A. thaliana* seedlings were weighed and lyophilized. GS were extracted with 80% methanol supplemented with sinigrin as internal standard. The extracted GS were purified on a Multiscreen 96 wells filter plate loaded with 45 μl DEAE-sephadex A25 anion exchange beads. The plate was washed once with distilled water, loaded with 200 μl of the GS extract and then washed with 80% methanol followed by two washes with distilled water. Elution was done by treating the plate with 100 μl of 3.5 mg/ml type H-1 aryl-sulfatase for an overnight reaction at room temperature, followed by a second elution with 100 μl distilled water.

### Glucosinolates Quantification

20 μl of GS solution were run on a Thermo Scientific HPLC system at 1 ml/min. The column was a Luna C18(2), 150 × 4.6 mm, 5 μm (Phenomenex, Torrance, CA, United States). The mobile phases were water (A) and acetonitrile (B), running time: 40 min. The gradient changed as follows: 1.5% B for 2.5 min, 20% B for 9 min, 20% B for 6 min, 95% B for 3 min and 1.5% B for 3 min. Afterward, the column was equilibrated at 1.5% B for 16.5 min. The GS were detected with a UV detector at 226 nm and the retention time for each GS was inferred by comparison to the respective pure analytical standard. In order to calculate molar concentrations of individual GS, relative response factors were used to correct for absorbance differences between the sinigrin standard and the other components of the extract (Brown et al., 2003). The amount of each GS was back calculated and expressed in nanomoles per milligram (nmols/mg) of fresh weight.

### RNA Isolation

Total RNA was isolated from 2-week-old liquid grown seedlings (see above) or 6-week-old soil grown Arabidopsis rosette leaves 48 h after inoculation with *B. cinerea* or half-strength grape juice as control. RNA was extracted with TRI-Reagent (Sigma-Aldrich, St. Louis, MO, United States), followed by treatment with TURBO DNA-free (Ambion, Waltham, MA, United States) to remove genomic DNA contamination. Gel electrophoresis, NanoDrop 2000 spectrophotometer (Thermo Scientific, Waltham, MA) and TapeStation Instrument (Agilent Technologies, Santa Clara, CA) were used to determine the quality and quantity of the RNA. Following extraction, the RNA was stored at –80°C for subsequent analysis.

### cDNA Library Construction and Sequencing

RNA samples (three biological replicates per sample) were subjected to poly-A selection in order to select for mRNA specifically, randomly fragmented and reverse transcribed to cDNA. Adaptors that contain sample-specific indexes were ligated to the fragments in order to tag each sample and size-specific magnetic beads were used for fragment size selection. Enrichment of adaptor-bound inserts was achieved by PCR amplification, thereby enabling sample quantification for loading onto the sequencer. Illumina HiSeq 2500 system (Illumina Inc., San Diego, CA, United States) was used to sequence 50 bp single reads.

Raw reads from each sample were processed by removing primer and adaptor sequences. The sequences quality per base was evaluated using FastQC v0.10.1, and low quality reads (*Q*-value < 30) were subsequently filtered out. The clean reads were aligned with TopHat v2.0.11 software against the *A. thaliana* genome (downloaded from the Ensembl Plants website) or the *Botrytis cinerea* genome (downloaded from the Broad Institute website) as references. Three mapping attempts were done in order to determine how many mismatches should be allowed per read (1, 3 or 5 mismatches) and the mapping files with up to 3 mismatches were used. The mapped reads were assigned to genes or transcripts based on the gene annotations file using HTSeq-count v.0.6.1 with the union mode.

### Analysis of Gene Expression and Functional Annotation

The differential gene expression was calculated by generating a matrix of normalized counts using the DESeq package v1.14.0. A threshold for false discovery rate (FDR) < 0.05 and fold change (FC) > 4 were used to determine significant differences in gene expression. We chose to analyze only the most highly differentially expressed genes, thus selecting a cutoff of FC > 4.

Functional annotation of differentially expressed genes was carried out using DAVID (Database for Annotation, Visualization and Integrated Discovery) bioinformatics resources v6.7, the MapMan bioinformatics tool v3.5.1R2 and the Blast2Go bioinformatics software v3.1.

### Quantitative Reverse-Transcription PCR Analysis

Total RNA (1 μg) was reverse transcribed with High Capacity cDNA Reverse Transcription Kit (Applied Biosystems, Waltham, MA, United States). Quantitative reverse transcription PCR was performed with the SYBR master mix and StepOne real-time PCR machine (Applied Biosystems, Waltham, MA, United States). The thermal cycling program was as follows: 95°C for 20 s and 40 cycles of 95°C for 3 s and 60°C for 30 s. Relative fold change in gene expression normalized to *Atef1a* (eukaryotic translation elongation factor 1 alpha) or *Bcactin* (*Bc1G_08198*) was calculated by the comparative cycle threshold 2^–ΔΔCt^ method. Primers used in qRT-PCR analysis of *A. thaliana* are listed in Supplementary Table 6 and for *B. cinerea* in Supplementary Table 7.

### Statistical Analysis

Student’s *t*-test was performed when data was normally distributed and the sample variances were equal. For multiple comparisons, one-way ANOVA was performed when the equal variance test was passed. Significance was accepted at *p* < 0.05. All experiments described here are representative of at least three independent experiments with the same pattern of results.

## Data Availability Statement

The data presented in the study are deposited in the Figshare online open access repository, accession number https://doi.org/10.6084/m9.figshare.19384043.v1.

## Author Contributions

OB and ML designed the experiments and wrote the article together. OB performed the majority of the experiments and analyzed the data, with assistance from ML. Both authors contributed to the article and approved the submitted version.

## Conflict of Interest

The authors declare that the research was conducted in the absence of any commercial or financial relationships that could be construed as a potential conflict of interest.

## Publisher’s Note

All claims expressed in this article are solely those of the authors and do not necessarily represent those of their affiliated organizations, or those of the publisher, the editors and the reviewers. Any product that may be evaluated in this article, or claim that may be made by its manufacturer, is not guaranteed or endorsed by the publisher.
