# Supplementary material for: IQD1 Involvement in Hormonal Signaling and General Defense Responses Against Botrytis cinerea
Source: Front Plant Sci. 2022 Apr 26;13:845140. doi: 10.3389/fpls.2022.845140 (PMC9087847; doi:10.3389/fpls.2022.845140)
Supplement: Supplementary file 1 [file Data_Sheet_7.pdf]

Supplementary data

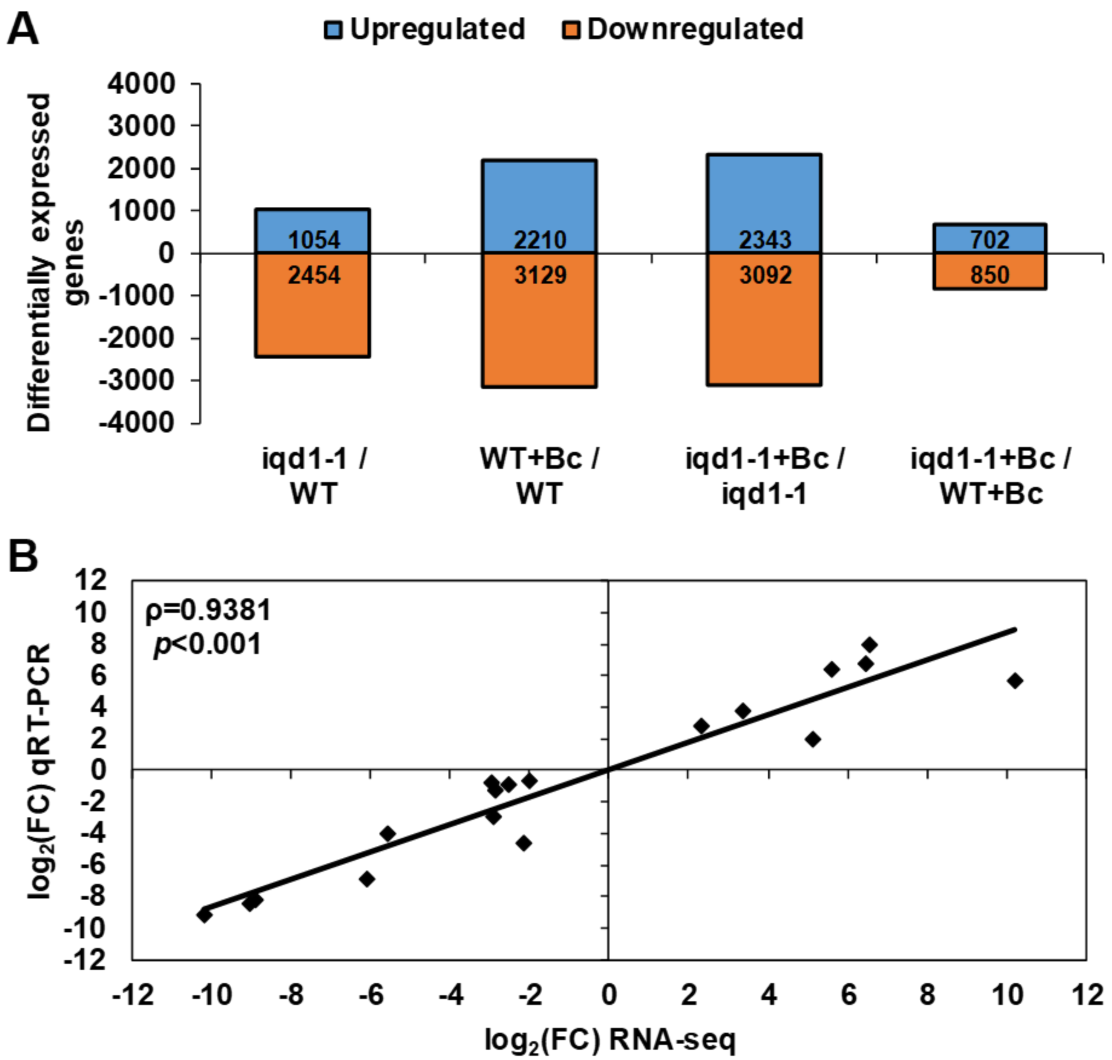

Figure S1. A. Differentially expressed *Arabidopsis thaliana* genes in mock and *B. cinerea* (Bc) infected plats**.** Shown are the number of upregulated and downregulated genes with more than four-fold difference between the following pairs: *iqd1-1* vs. WT, infected WT vs. WT, infected *iqd1-1* vs. *iqd1-1*, infected *iqd1-1* vs. infected WT. **B.** Correlation of qRT-PCR and RNA-Seq results. Expression ratios of 18 representative genes were determined using qRT-PCR for iqd1-1/WT. Each RNA-Seq value and qRT-PCR value (mean of 4 repeats) were log_2 transformed and plotted against each other for comparison. Correlation analyses were done with Spearman's Rho test.

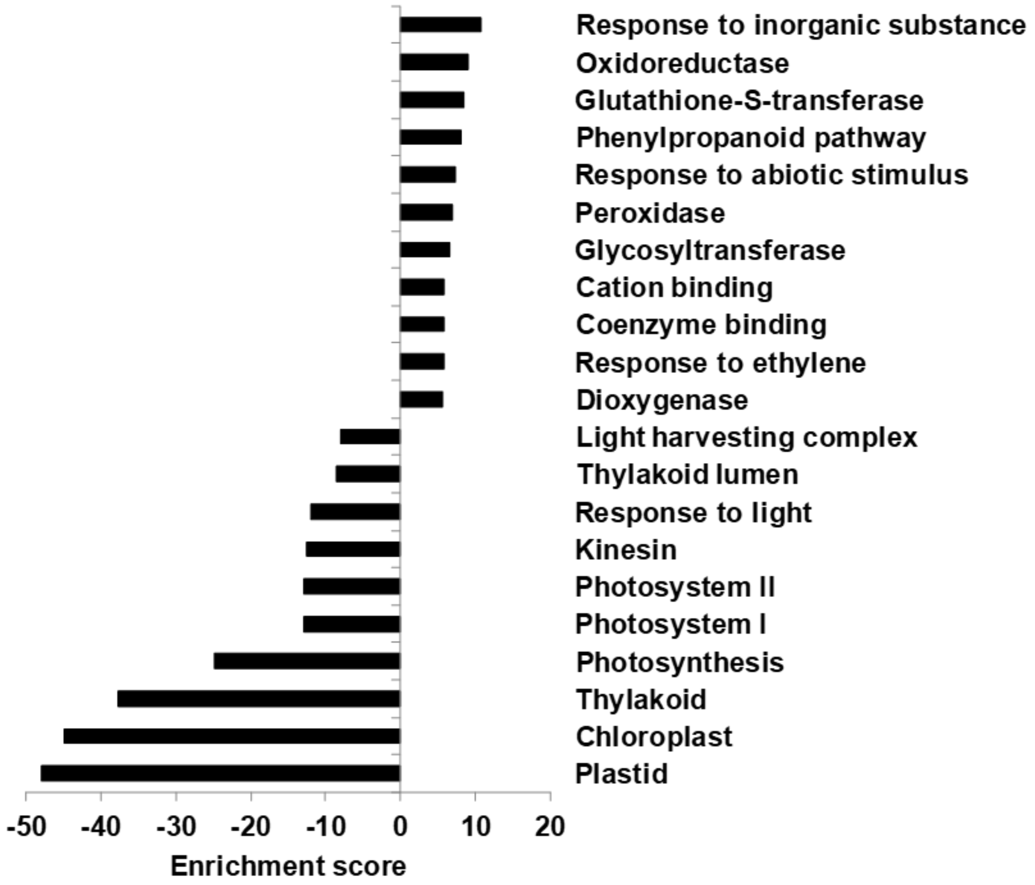

Figure S2. Differentially expressed clusters in infected vs. mock inoculated WT plants. Enriched annotation terms of functional-related genes were grouped into clusters using the DAVID bioinformatics resources website. Positive enrichment scores denote upregulated clusters in infected WT while negative values denote upregulated clusters in mock treated WT plants.

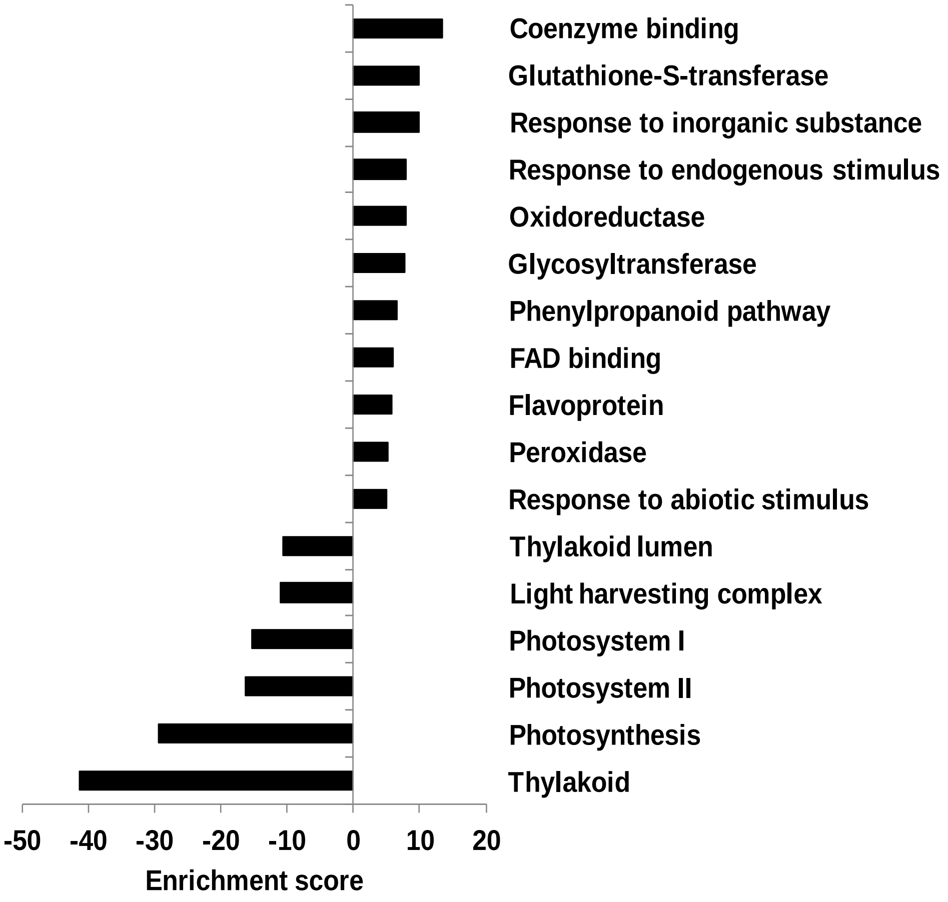

Figure S3. Differentially expressed clusters in infected vs. mock inoculated iqd1-1 plants. Enriched annotation terms of functional-related genes were grouped into clusters using the DAVID bioinformatics resources website. Positive enrichment scores denote upregulated clusters in infected iqd1-1 while negative values denote upregulated clusters in mock treated iqd1-1 plants.

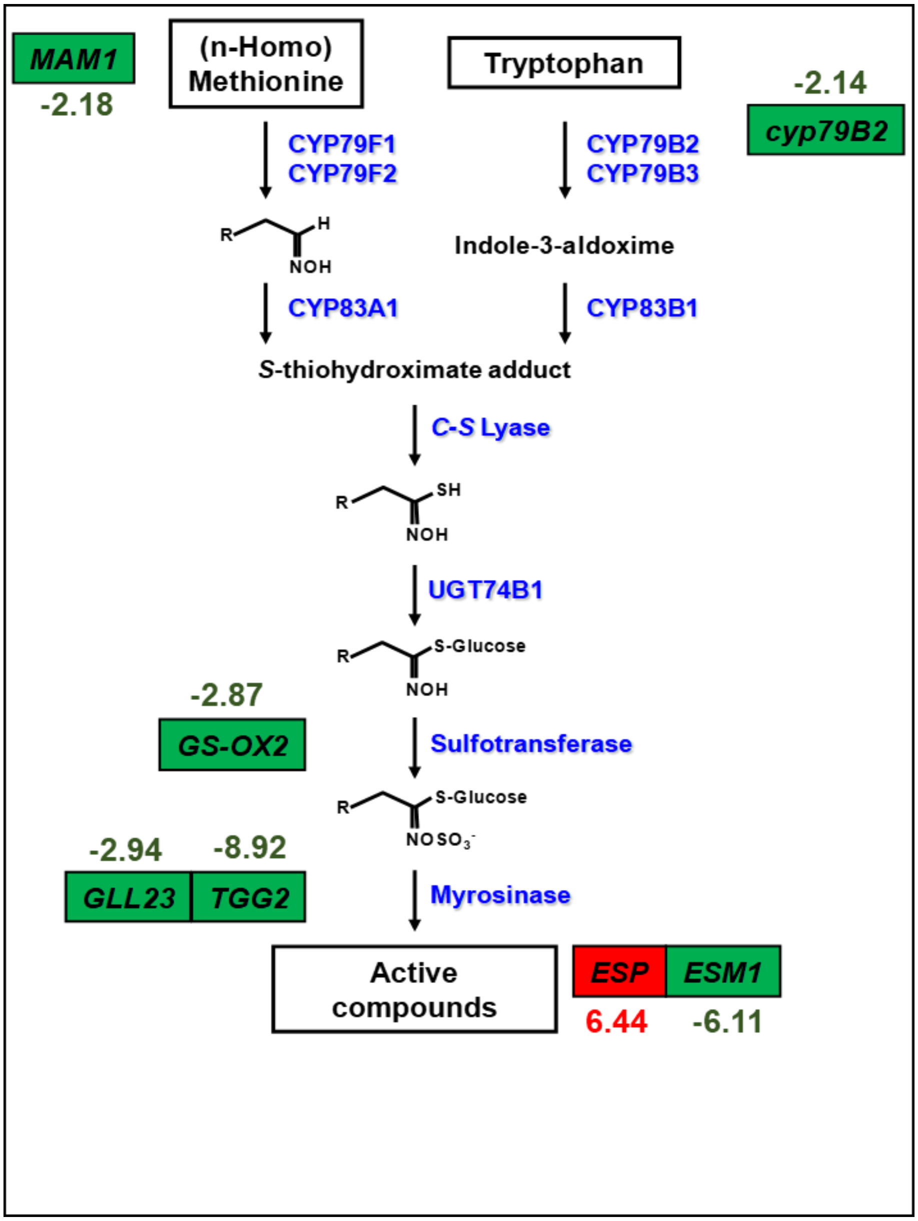

Figure S4. Differentially expressed GS related genes in iqd1-1 compared to WT. Schematic overview of important genes involved in glucosinolate biosynthesis. Upregulated genes in iqd1-1 are in red boxes and downregulated genes are in green boxes (numbers represent fold change).

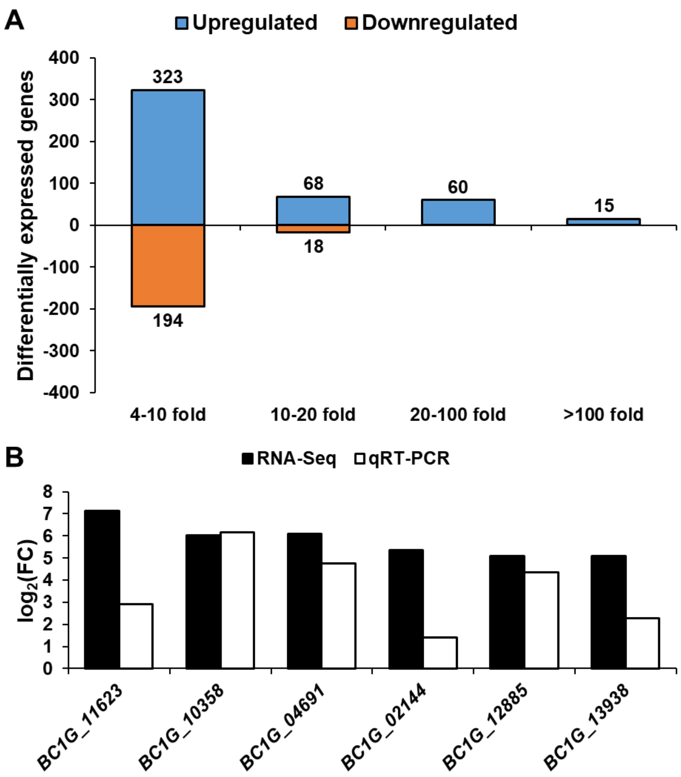

Figure S5. A. Differentially expressed Botrytis cinerea genes when infecting iqd1-1 compared to WT. Shown are the number of upregulated (positive values) and downregulated (negative values) genes with more than four-fold difference between B. cinerea infecting iqd1-1 as compared to infecting WT. **B**. qRT-PCR validation of RNA-Seq results. Comparison of B. cinerea candidate genes expression levels after infection of iqd1-1 plants vs. WT.

Table S1. Reads of each dataset and mapping results

Read type	Ws-0	*iqd1-1*
Total reads	45,881,751	47,458,716
Mapped reads*	6,359,210 (13.86%)	3,659,067 (7.71%)
Uniquely mapped reads*	3,129,135 (6.82%)	2,477,345 (5.22%)
Multiple mapped reads*	3,230,075 (7.04%)	1,181,722 (2.49%)
Unmapped reads*	39,522,541 (86.14%)	43,799,649 (92.29%)
Uniquely counted reads**	2,488,753 (79.535%)	1,955,746 (78.945%)
Multiple counted reads**	125 (0.004%)	74 (0.003%)
Uncounted reads**	640,257 (20.461%)	521,525 (21.052%)

Table S2. Summary of mapped and counted reads of RNA from *B. cinerea* and mock infected tissues.

Read type	WT	*iqd1-1*	WT+Bc	*iqd1-1*+Bc
Total reads	49,778,547	31,852,780	45,881,751	47,458,716
Mapped reads*	48,275,235 (96.98%)	29,779,164 (93.49%)	40,527,351 (88.33%)	42,480,297 (89.51%)
Uniquely mapped reads*	44,541,844 (89.48%)	26,335,879 (82.68%)	26,207,656 (57.12%)	35,043,516 (73.84%)
Multiple mapped reads*	3,733,391 (7.50%)	3,443,285 (10.81%)	14,319,695 (31.21%)	7,436,781 (15.67%)
Unmapped reads*	1,503,312 (3.02%)	2,073,616 (6.51%)	5,354,400 (11.67%)	4,978,419 (10.49%)
Uniquely counted reads**	43,129,103 (96.828%)	25,238,861 (95.835%)	24,014,417 (91.631%)	33,296,296 (95.014%)
Multiple counted reads**	636,503 (1.429%)	433,225 (1.645%)	357,735 (1.365%)	556,491 (1.586%)
Uncounted reads**	778,591 (1.746%)	664,718 (2.524%)	1,837,157 (7.010%)	1,190,779 (3.398%)

Table S3. *Arabidopsis thaliana* homozygous mutants verification primers

Mutant	Primer name	Sequence
IQD1^{OXP}	H30 For	CGGAGGCGGAGAAAGTTATTG
	H30 Rev	CACAGGCAAAGCATTCACGAC
NahG	NahG1 f	CTGCCGCTACTCCCATATCCA
	NahG1 r	TCGGCTTCGGCTCGCTAC
npr1-1	npr1-1 f	AGGCACTTGACTCGGATGAT
	npr1-1 r	ATGCACTTGCACCTTTTTCC
aos (Salk_017756)	Salk_017756 LP	CGAGAAATTAACGGAGCTTCC
	Salk_017756 RP	CTAACCGGAGGCTACCGTATC
coi1 (Salk_095916)	Salk_095916 LP	TCACCGACCTTCACAGATACC
	Salk_095916 RP	TGGTTCAAGATTGATTCCGAG
jar1-1	jar1-1 f	CAGTGTGTGTGTTTTTGATCATAAGCT
	jar1-1 r	CAAATTTAAACTATACCTGTTTCTGAAGG
ein2-1	ein2-1 f	CCAGAGGAAAGAGAGTTGGATGTAAAGTACTCTACCG CT
	ein2-1 r	CGCCATCTTTGTTTCAACAATCAGATCC
eto1-1	eto1-1 f	GCAACACAACTTGACCCTCTT
	eto1-1 r	GGGAGAATCCCTCAGAAAGG
pad3-1	pad3-1 f	CAAAGACATCGGGATGGCAC
	pad3-1 r	AGCCTTTAGCACAAGATCCACG
cyp79B2	cyp79B2 F	CTCGTTCAAGAATCCGACATCC
	cyp79B2 R	TCTCCGGTTTAAAGCAAAGTGG
cyp79B3	cyp79B3 F	AAGGCAATCCACCAATATCCG
	cyp79B3 R	TCGTGGTCAACATGCTTTATGC

Table S4. LC-MS/MS conditions

Time (minutes)	Solvent A (%), Water + 0.1% acetic acid	Solvent B (%), Methanol
0	80	20
1	80	20
12	4	96
20	4	96
20.1	80	20
25	80	20
Additional parameters		
Column temperature (°C)		40
Injection volume (µl)		5
Flow rate (ml/min)		0.25

Table S5. MRM parameters.

Compound	Fragmentor voltage (V)	Collision energy (eV)	MRM transitions
Abscisic acid	80	10	263→219
		10	263→204
Abscisic acid D6	80	10	269→225
		10	269→159
Jasmonic acid	80	10	209→59
Jasmonic acid D5	80	10	213→61
Salicylic acid	80	15	137→93
		15	137→65
Salicylic acid D4	80	15	141→97

Table S6. *Arabidopsis thaliana* qRT-PCR primers

Gene	Primer name	Sequence
ef1a	Ef1a SYBR f1	TGAGCACGCTCTTCTTGCTTTCA
	Ef1a SYBR r1	GGTGGTGGCATCCATCTTGTTACA
nsp4	NSP4 f1	TCAAGTTTGAGTATGTCAATGGTTCTC
	NSP4 r1	CAATCTCAAACTCTTCAACTCCTAGCT
esp	ESP f1	CTACAGGAGCGAAACCTTCC
	ESP r1	GATCAGGCCATACCTCACCT
cml42	CML42 f1	TCGGATCTCGCCGAGGCGTT
	CML42 r1	ACGCGACCATCTTGATTCCGGT
erf114	ERF114 f1	CAAGTTGCGCCTACTCATCA
	ERF114 r1	TTTTGGGTCTCGGATTTCAG
At4G24420	AT4G24420 f1	ACGCTGATGAGAAGGTGATGCT
	AT4G24420 r1	GGGAGTAGGGTTGGCCTTGA
tip2-3	TIP2-3 f1	TTCTATTGGATTGCTCAGTGTCTTG
	TIP2-3 r1	GGTACGCTCTTGCCATTAGTAACA
At4G22490	AT4G22490 f1	AGTGTTGCACTGCCCTCAATG
	AT4G22490 r1	CAACGGAATCGGCCTGAA
sr1	SR1 SYBR f2	TTTGACGCCCAGGGATAGTT
	SR1 SYBR r2	GGACTGTTGATAGTTTTGCTTTTGTC
iqd24	IQD24 f1	TCCGCTTTTCGTGGCTACTT
	IQD24 r1	CCCTTCACCAACGCTTGAA
iqd31	IQD31 f1	TTCAAGATCATTCATTATCTGATGCA
	IQD31 r1	GCCGAGCCAAGTAACCTCTAAA
esm1	ESM1 f1	TCGTAGGATTGCGACAGG
	ESM1 r1	CCTGAGCCTTCTCTGTGTTG
tgg2	TGG2 f1	CCGCAAGGCCATCAAGGAGAAG
	TGG2 r1	AATCTGACGGTGTAGCCGTTGC
mam1	MAM1 f1	CGGCTGAAAGAGTTGGGATA
	MAM1 r1	ATGCCTTCAAATCAGCATCC
fmo gs-ox2	GS-OX2 f1	GCCGTTGTTGTTTGCAGTGGAC
	GS-OX2 r1	ATTTCCGATGACCACCACCACCTC
gll23	GLL23 f1	CAGACTTCCTCGCTAAATTCATGA
	GLL23 r1	CTCCACGTGAGACATTGACGTT
At1G63880	AT1G63880 f1	TGCATTCTAGCCAGCTCGAGTA
	AT1G63880 r1	TCCGGGAGTTGCTTCAAATT
At1G66100	AT1G66100 f1	TGTCTTGACATTAGTGAGTCGACATG
	AT1G66100 r1	TCAGTGACAGCATCACCTGTGTT
abcc11	ABCC11 f1	TTCAAGGTTGGGCATGATCTATC
	ABCC11 r1	ACCCAAGCTGGATCACCTTCT
cyp705A27	CYP705A27 f1	CTTATTGTGGAGCTTTTCCTTGGA
	CYP705A27 r1	TGGTTAATGAGTTCGGCCATT
cyp71A24	CYP71A24 f1	ATCTTGGATGTTTTTGTGGGTGAT
	CYP71A24 r1	TGGTGGCATAGTAGCTCTGTCATT
cyp76C6	CYP76C6 f1	TCGAACACCTACTTCTCGATATGTTT
	CYP76C6 r1	TAACTCTGCCATTGCCCATTC
cyp82C2	CYP82C2 f1	AATCTACCTGCCTGGCACTG
	CYP82C2 r1	GAGAAATGGCCCATGTAAGG
At1G66540	AT1G66540 f1	GATTGATCACTTGCTTTCTTTGCA
	AT1G66540 r1	CGCAAGTATAAGAGAAAGCATGGTT
cyp81K2	CYP81K2 f1	GTCGCTTGCTGTTGGAGCAT
	CYP81K2 r1	ACGCAGGCCTCATGTCTATGT
cyp76C5	CYP76C5 f1	CACCTACTTCTCGATATGTTTCTAGCA
	CYP76C5 r1	CTCTGTCATTGCCCATTCCA
cyp72A11	CYP72A11 f1	TCAAGACTCATGGGAGGACTTTC
	CYP72A11 r1	TGTGATTTGCTCAGGATCCATT

Table S7. *Botrytis cinerea* qRT-PCR primers.

Gene	Primer name	Sequence
Bcactin (*Bc1G_08198*)	08198 F	CCCAATCAACCCAAAGTCCAACAG
	08198 R	CAAATCACGACCAGCCATGTC
Bc1G_11623	11623 F	GTTTGCATCTACCGGATCGT
	11623 R	AAACACAGCACCACCATTGA
Bc1G_10358	10358 F	GGCGAGCAAAACGCTCTAT
	10358 R	GGTGAGAGGAGGAGCTTGTG
Bc1G_04691	04691 F	CATAACCGAAAACGGCACTT
	04691 R	GGCCCACTCAAAGTTATCCA
Bc1G_02144	02144 F	GTTGATGCCACCTGGAAACT
	02144 R	GGGGAGTCAAATGCGTAGAA
Bc1G_12885	12885 F	TTCACCAAAGTCCCTCAACC
	12885 R	AACCGAGGCTACCATCATTG
Bc1G_13938	13938 F	CTCTTTTGCACTGCTTGCTG
	13938 R	TTCCAGGTCTCTCCACCATC
